# A transcription factor network controls cell migration and fate decisions in the developing zebrafish pineal complex

**DOI:** 10.1242/dev.131680

**Published:** 2016-07-15

**Authors:** Sataree Khuansuwan, Joshua A. Clanton, Benjamin J. Dean, James G. Patton, Joshua T. Gamse

**Affiliations:** Department of Biological Sciences, Vanderbilt University, Nashville, TN 37235, USA

**Keywords:** Flh, Nr2e3, Parapineal organ, Pineal complex, Tbx2b, Zebrafish

## Abstract

The zebrafish pineal complex consists of four cell types (rod and cone photoreceptors, projection neurons and parapineal neurons) that are derived from a single pineal complex anlage. After specification, parapineal neurons migrate unilaterally away from the rest of the pineal complex whereas rods, cones and projection neurons are non-migratory. The transcription factor Tbx2b is important for both the correct number and migration of parapineal neurons. We find that two additional transcription factors, Flh and Nr2e3, negatively regulate parapineal formation. Flh induces non-migratory neuron fates and limits the extent of parapineal specification, in part by activation of Nr2e3 expression. Tbx2b is positively regulated by Flh, but opposes Flh action during specification of parapineal neurons. Loss of parapineal neuron specification in Tbx2b-deficient embryos can be partially rescued by loss of Nr2e3 or Flh function; however, parapineal migration absolutely requires Tbx2b activity. We conclude that cell specification and migration in the pineal complex are regulated by a network of at least three transcription factors.

## Abstract (precis)

**Summary:** Cell fate specification and migration in the zebrafish pineal complex are regulated by a network of at least three transcription factors: Tbx2b, Flh and Nr2e3.

## INTRODUCTION

The development of the vertebrate nervous system proceeds via a series of well-conserved steps during which the correct numbers of neurons are specified and assume appropriate positions in order to establish precise connectivity patterns (for a review, see Guillemot, 2007). However, the exact mechanisms that fine-tune the specification of the myriad of neuronal subtypes remain unknown. This is exemplified by cell-type specification in vertebrate retinal development, during which one glial and six neuronal cell types derive from a common group of progenitors (Turner and Cepko, 1987). Although numerous genes that govern this process have been uncovered, the precise mechanisms that produce different neuron types remain elusive (for a review, see Cepko, 2014). To elucidate these complex developmental mechanisms, it is useful to study simpler systems. One such system is the pineal complex of the zebrafish brain. The zebrafish pineal complex is composed of a centrally located pineal organ and an asymmetrically positioned parapineal organ that, together with the flanking habenular nuclei, form a region of the forebrain called the dorsal diencephalon. Although they are derived from the same anlage, the pineal and parapineal organs comprise distinct neuronal types and perform different functions. The zebrafish pineal organ, which is directly photoreceptive, is made up of rod and cone photoreceptors and associated projection neurons that are non-migratory (Concha and Wilson, 2001; Halpern et al., 2003; Mano and Fukada, 2007); its chief function is to secrete melatonin, a hormone that synchronizes physiological functions with circadian stimuli. The parapineal organ is derived from progenitor cells that originally intermingle with pineal organ progenitors. Following cues from left-sided Nodal signaling, these cells later migrate unilaterally to the left side of the brain (Concha et al., 2003; Snelson et al., 2008b). Through an unknown mechanism, this left-sided migration is an important driver of asymmetry within the habenular nuclei in the dorsal diencephalon. The left habenula, which is innervated by parapineal neurons, has denser neuropil and more robust expression of the gene Kctd12.1 compared with the right habenula (Concha et al., 2003; Gamse et al., 2003). Left habenular neurons are chiefly responsive to visual stimuli, in contrast to the right habenular neurons, which are more highly activated by olfactory stimuli (Dreosti et al., 2014). Ablation of the parapineal organ causes the left habenula to adopt gene expression patterns more characteristic of the right, and to respond more readily to odor rather than light (Dreosti et al., 2014; Gamse et al., 2003).

The relative numbers of neuronal cell types produced during pineal complex development are very consistent across embryos, suggesting the existence of a robust molecular mechanism that governs cell specification in the pineal complex. Indeed, within the pineal organ, BMP and Notch activities regulate the decision between photoreceptor and projection neuron cell fates (Cau et al., 2008; Quillien et al., 2011). However, assignment of pineal versus parapineal cell fates seems to be largely governed by two transcription factors: Flh and Tbx2b (Masai et al., 1997; Snelson et al., 2008b). Flh, a homeodomain-containing transcription factor (recently renamed Noto), was initially thought to be solely required for the formation of the pineal organ. In Flh mutants, neurogenesis in the pineal organ stalls at about 18 h post-fertilization (hpf), resulting in large deficits in all subtypes of pineal cells (Masai et al., 1997); despite the loss of these cells, the parapineal organ can still form in Flh mutants (Snelson et al., 2008a). By contrast, parapineal development is dramatically affected in Tbx2b mutants, which have fewer parapineal neurons; the few that do differentiate fail to migrate away from the largely normal pineal organ (Snelson et al., 2008b). Thus, the function of these two transcription factors initially appeared to be complementary, with Tbx2b specifying migratory parapineal neurons and Flh controlling the formation of the non-migratory pineal cell types.

Despite their abilities to specify different cell types, *flh* and *tbx2b* expression domains in the epithalamus largely overlap during most of pineal complex development (Snelson et al., 2008b). In addition, two contradictory findings raised questions about possible regulation of *tbx2b* by Flh (Cau and Wilson, 2003; Snelson et al., 2008a). To clarify the relationship between Flh and Tbx2b, we analyzed the expression of these genes at a more appropriate time point (10 somite stage, ss) and found that Flh does indeed regulate *tbx2b*. In this article, we show that Flh represses parapineal fate and promotes pineal fate. We also show that the orphan nuclear receptor and transcription factor Nr2e3 is positively regulated by Flh and is partially responsible for the suppression of parapineal fate. Loss of Nr2e3 results not only in more parapineal neurons, but also reduced pineal rod photoreceptors. We establish that Tbx2b is unequivocally required for parapineal cell migration and antagonizes Flh-mediated repression of parapineal cell fate. We conclude that Flh, Nr2e3 and Tbx2b, through a complex regulatory mechanism, establish an equilibrium that generates the correct number of pineal complex cells and ensures proper migration of parapineal neurons.

## RESULTS

### Reduced expression of *tbx2b* in pineal anlage of Flh mutants

**Fig. 1.**
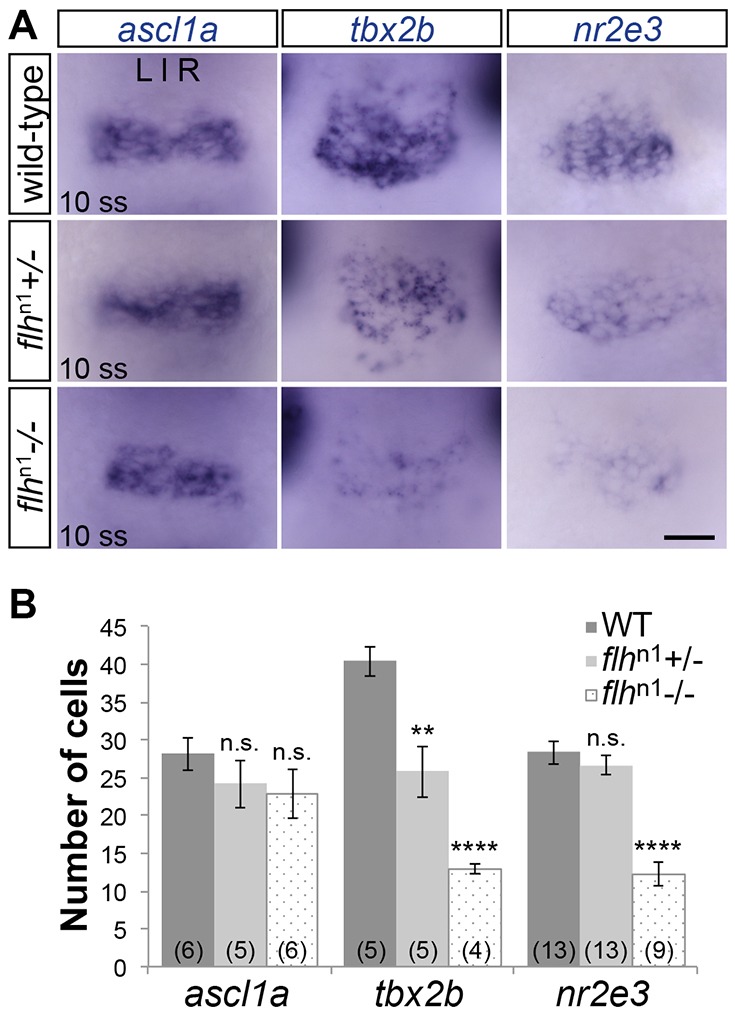
**Decreased expression of *tbx2b* and *nr2e3* in Flh mutants.** (A) *In situ* hybridization showing dorsal views of *ascl1a*, *tbx2b* and *nr2e3* expression in the pineal anlage of WT, *flh*^n1+/−^ or *flh*^n1−/−^ embryos at 10 ss (14 hpf). Scale bar: 30 µm. (B) Quantification of the number of cells expressing *ascl1a*, *tbx2b* or *nr2e3* at 10 ss under the indicated genetic backgrounds; mean±s.e.m. and number of samples (*n*) are shown. ****P<0.0001, **P<0.01, *P*>0.05 (n.s.), one-way ANOVA with Dunnett's post-hoc analysis.

In order to determine conclusively whether *tbx2b* expression is affected by Flh, we performed *in situ* hybridization at 10 ss (14 hpf). At this stage, the presumptive pineal complex anlage is contiguous at the midline and pineal neurogenesis still appears to be developing normally in *flh*^n1^ homozygous mutants, as indicated by the expression of *achaete-scute family bHLH transcription factor 1a* (*ascl1a*), a transcription factor often expressed in newly specified neurons. Expression of *ascl1a* was comparable between wild-type (WT), *flh*^n1+/−^ and *flh*^n1−/−^, suggesting that pineal complex neurogenesis is not drastically affected by loss of Flh at this stage (Fig. 1A). Compared with WT, *tbx2b* expression, as well as the number of cells expressing *tbx2b*, were reduced in *flh*^n1+/−^ and *flh*^n1−/−^ in a dosage-dependent manner, consistent with the idea that *tbx2b* is activated by and genetically downstream of Flh (Fig. 1A,B).

### Flh inhibits specification of parapineal neurons in a dosage-dependent manner

**Fig. 2.**
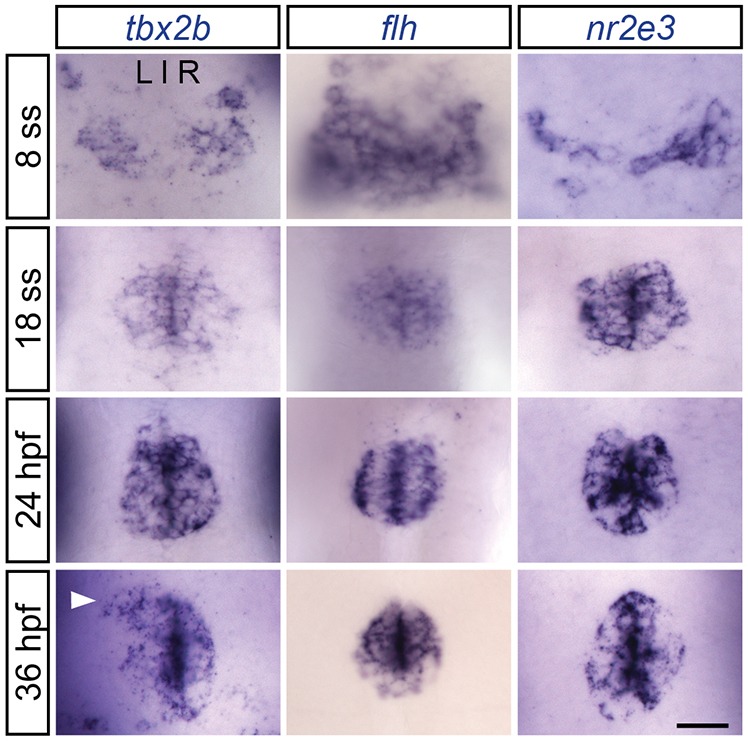
***tbx2b, flh* and *nr2e3* are expressed early during parapineal development.** Dorsal views of *in situ* hybridizations of *tbx2b*, *flh* and *nr2e3* in the pineal complex at the indicated stages. At 8 ss (13 hpf), the pineal anlage is in the process of fusing at the midline. Parapineal specification occurs around 15-18 hpf. By 24 hpf, *tbx2b-*expressing parapineal precursors begin to coalesce at the anterior-most region of the pineal anlage. By 36 hpf, a group of parapineal neurons can be observed as a distinct *tbx2b-*positive, *flh/nr2e3-*negative population that has migrated away from the pineal organ (arrowhead). Scale bar: 30 µm.

**Fig. 3.**
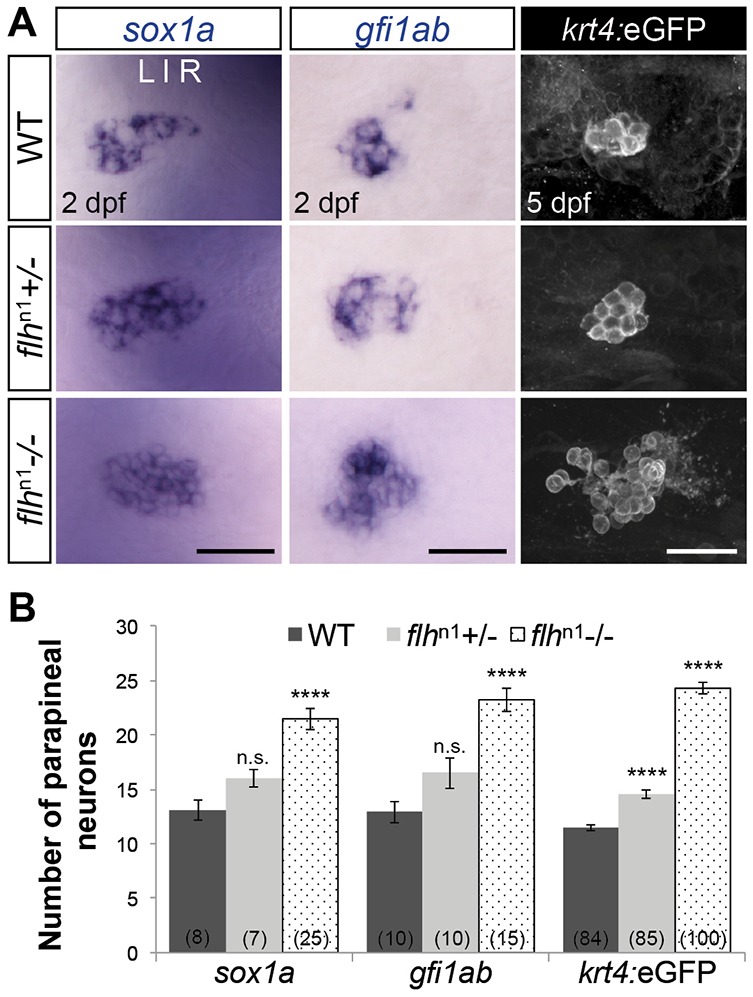
**Flh inhibits specification of parapineal neurons in a dosage-dependent manner.** (A) Dorsal views of *in situ* hybridizations of *sox1a* and *gfi1ab* and antibody labeling of *krt4*:eGFP in the epithalamus under the indicated genetic backgrounds and stages. Scale bars: 30 µm. (B) Quantification of the number of cells expressing different markers of parapineal neurons; mean±s.e.m. and number of samples (*n*) are shown. ****P<0.0001, *P*>0.05 (n.s.), one-way ANOVA with Dunnett's post-hoc analysis.

Similar to *tbx2b*, *flh* is expressed in the pineal complex throughout parapineal development (Fig. 2). However, unlike *tbx2b*, *flh* is not expressed in the parapineal organ itself (Fig. 2). A previous publication reported that Flh and Tbx2b act in separate genetic pathways with Flh specifying pineal cell types and Tbx2b governing parapineal cell fate (Snelson et al., 2008a). However, if Flh is a positive regulator of *tbx2b* expression, and Tbx2b mutants have a reduced number of parapineal neurons (Snelson et al., 2008b), then loss of Flh should also lead to a reduced number of parapineal neurons. However, using three different markers of parapineal neurons (*sox1a*, an early differentiation marker; *gfi1ab*, a late differentiation marker; and Tg[*krt4:eGFP*]^sqet11^, a transgenic line that expresses eGFP in parapineal neurons by 5 days post-fertilization, dpf), we found that homozygous Flh mutants, *flh*^n1^, exhibited about double the number of parapineal neurons compared with WT (Fig. 3). Furthermore, we found that this increase was dependent on the levels of Flh. Larvae with only one mutant copy of Flh (*flh*^n1+/−^) had an intermediate number of parapineal neurons, more than WT but less than *flh*^n1^ homozygous larvae (Fig. 3B). We conclude that Flh inhibits parapineal neuronal specification in a dosage-dependent manner.

In addition to pineal neurogenesis, Flh plays a crucial role during the formation of the notochord, an important midline structure during zebrafish embryonic development (Talbot et al., 1995). To investigate whether or not the observed increase in parapineal neuron number is a secondary effect of having a compromised midline, we looked at *ntl* morphants, which lack notochord tissue (Halpern et al., 1993), and found no difference in parapineal neuron numbers between *ntl* morphants (11.2±1.8, *n*=10; here, and throughout the main text, values given are mean±s.d.) and non-injected controls (NICs) (10.8±1.6, *n*=10, *P*=0.650). Thus, simply having midline defects did not lead to greater numbers of parapineal neurons being specified. This suggests that Flh regulates the number of parapineal neurons directly and not via formation of the notochord. Furthermore, no significant changes were observed in the mitotic index within the pineal complex between *flh*^n1^ and their siblings (*flh*^n1+/−^ and WT) during parapineal development, suggesting that the increase in parapineal neurons is not due to an overall increase in, or a shift in the timing of, cell division (Fig. S1).

### Tbx2b and Flh act in parallel during specification of parapineal neurons

**Fig. 4.**
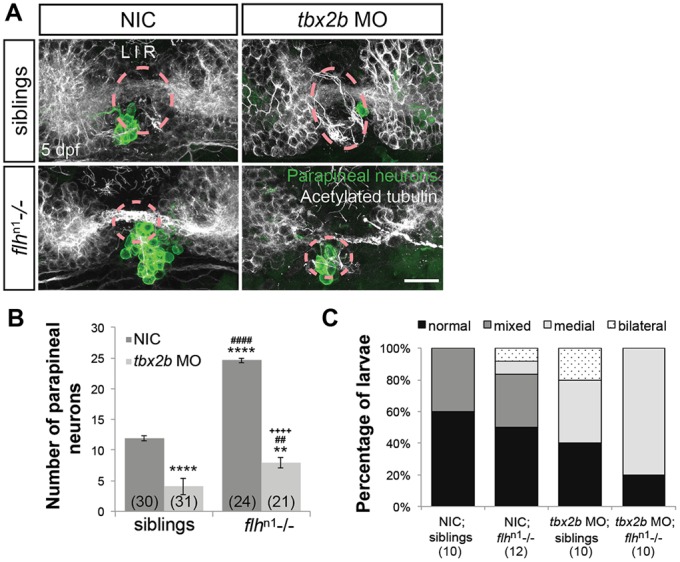
**Flh acts in parallel with Tbx2b to specify parapineal fate, but does not govern migration.** (A) Dorsal views of antibody labeling of parapineal neurons (*krt4*:eGFP+) as well as axons and dendrites (acetylated tubulin^+^) in the epithalamus at 5 dpf. The pineal organ regions are marked by dashed circles. Scale bar: 30 µm. (B) Quantification of the number of parapineal neurons in siblings (WT and *flh*^n1+/−^) or *flh*^n1−/−^ mutants that were either non-injected (NIC) or injected with *tbx2b* morpholinos; mean±s.e.m. and number of samples (*n*) are shown. Loss of Tbx2b function suppresses the *flh*^n1−/−^ supernumerary parapineal specification phenotype, as well as migration of parapineal neurons. ****P<0.0001, **P<0.01, *P*>0.05 (n.s.), one-way ANOVA with Tukey's post-hoc analysis, comparisons with NIC;siblings (*), comparisons with *tbx2b* MO;siblings (#), and a comparison with *flh*^n1−/−^ (+) are shown. (C) Quantification showing the percentages of larvae that display normal, bilateral, mixed (some neurons migrate and others do not), or medial (all neurons remain near the midline) parapineal-migration phenotypes and number of samples (*n*).

*tbx2b* expression is reduced but not totally lost in *flh*^n1^ homozygous mutants (Fig. 1). Given the unexpected increase in parapineal neuron numbers in *flh*^n1^, we wanted to determine what role residual *tbx2b* might play in the *flh*^n1^ mutant phenotype. Therefore, we further knocked down Tbx2b levels in *flh*^n1^ mutant background by morpholino injections and found a greater than 65% reduction in parapineal neuron numbers in both *flh*^n1^ mutants and their siblings (Fig. 4A,B). If Tbx2b directly specifies parapineal fate independently of Flh, we would expect *flh*^n1^/*tbx2b* morphants to have a similar number of parapineal neurons as siblings/*tbx2b* morphants. However, despite the depletion of Tbx2b function, a near wild-type number of parapineal neurons remained in *flh*^n1^/*tbx2b* morphants (8.0±4.1, *n*=21 versus 11.9±2.3, *n*=30 for NICs/siblings) (Fig. 4B). These data suggest that Tbx2b does not directly specify parapineal fate; rather, it functions to prevent Flh from repressing parapineal fate.

**Fig. 5.**
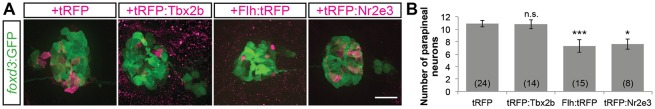
**Mosaic overexpression of Tbx2b does not induce additional parapineal neurons; mosaic overexpression of Flh or Nr2e3 suppresses the number of parapineal neurons.** (A) Representative images of 2 dpf antibody-labeled pineal complexes (*foxd3*:GFP+) with the indicated protein mosaically overexpressed. Scale bar: 30 µm. (B) Quantification of the number of parapineal neurons in Tg[*foxd3*:GFP] ^zf10^; Tg[*cfos*:*gal4vp16*]^s1145t^ double transgenic embryos injected with pDestTol2CG2(*4nrUAS:tagRFP-T:polyA*) (tRFP), pDestTol2CG2(*4nrUAS:tagRFP-T:tbx2b*) (tRFP:Tbx2b), pDestTol2CG2(*4nrUAS:flh:tagRFP-T*) (Flh:tRFP), or pDestTol2CG2(*4nrUAS:tagRFP-T:nr2e3*) (tRFP:Nr2e3); mean±s.e.m. and number of samples (*n*) are shown. ***P <0.001, *P<0.05, *P*>0.05 (n.s.), one-way ANOVA with Dunnett's post-hoc analysis, comparisons with tRFP shown.

To test directly whether Tbx2b can induce parapineal fate, we mosaically overexpressed a tRFP-tagged version of Tbx2b (tRFP:Tbx2b) specifically within the pineal complex anlage during development using the GAL4/UAS system. We found that overexpression of tRFP:Tbx2b does not correlate with an increase in parapineal neurons at 2 dpf (Fig. 5B). Conversely, mosaic overexpression of Flh:tRFP in a similar number of pineal complex cells resulted in reduced numbers of parapineal neurons (Fig. 5B).

Together, our gene expression, loss-of-function, and mosaic overexpression data suggest that a complex interplay between Tbx2b and Flh regulates parapineal neuron number. First, Flh governs, directly or indirectly, *tbx2b* expression early during pineal complex development. Second, Flh can effectively suppress parapineal cell fate. Third, Tbx2b is required for parapineal development, but cannot directly induce parapineal cell fate. It should be stressed that the effect of Flh on parapineal development is not ‘all or none’ and is linked to Tbx2b function.

### Flh promotes specification of non-migratory pineal cell types

We have established that Flh acts in a dosage-dependent manner during parapineal specification. To test whether Flh also functions during specification of pineal cell types, we compared the numbers of rods, cones, and projection neurons between wild-type and *flh*^n1+/−^ larvae. Although we observed a 13.8% (11.5 cells) reduction in the total number of pineal complex cells in Flh^+/−^ mutants (determined by ToPro staining of nuclei), we detected a much greater reduction (34.4%, 28.6 cells) in the combined number of rods (Rhodopsin), red/green cones (Arr3a), and projection neurons (HuC/D; also known as Elavl3/4) by 4 dpf (Table 1). Therefore, in addition to its role in the progression of pineal neurogenesis and inhibition of parapineal neuron specification, Flh is also required for neuronal specification of all non-migratory pineal cell subtypes.

**Table 1.**
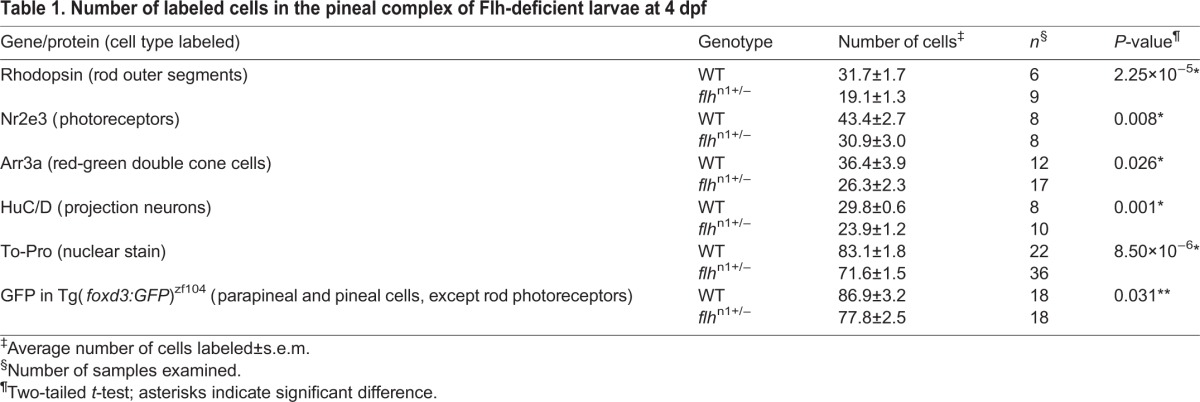
**Number of labeled cells in the pineal complex of Flh-deficient larvae at 4 dpf**

### Nr2e3 promotes pineal rod photoreceptor specification

**Fig. 6.**
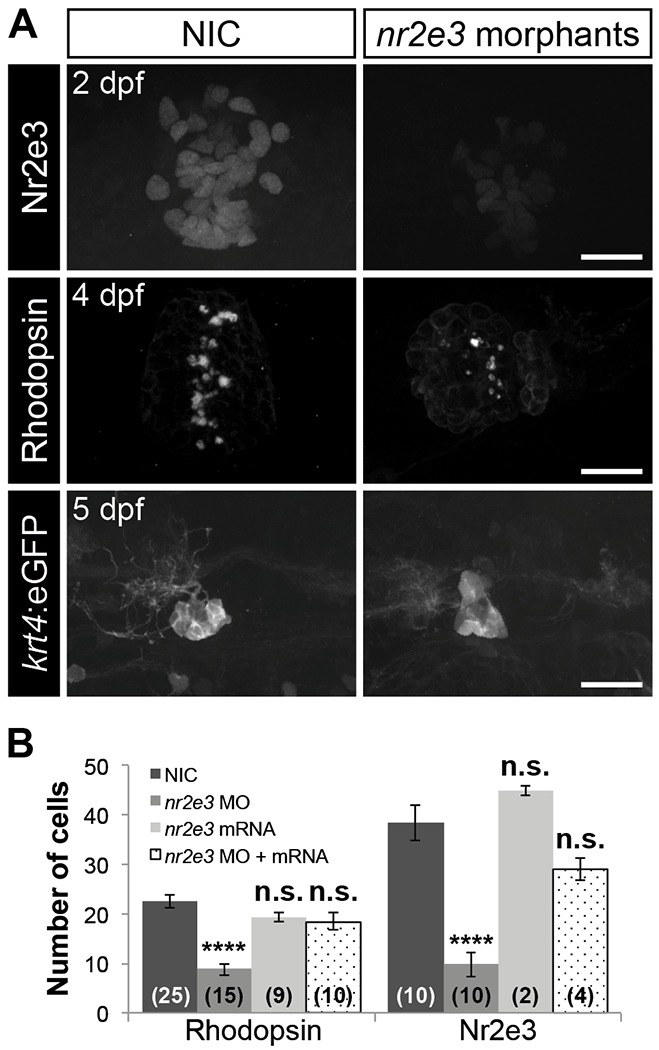
**Nr2e3 inhibits specification of parapineal neurons and is necessary for pineal rod photoreceptors specification.** (A) Representative images of antibody labeling of Nr2e3, Rhodopsin and *krt4*:eGFP in the pineal complexes of non-injected controls (NIC) and *nr2e3* splice morphants at the indicated stages. Dorsal views. Scale bars: 30 μm. (B) Quantification of the number of Rhodopsin- or Nr2e3-positive cells at 2 dpf in NICs, *nr2e3* splice morphants, *nr2e3* mRNA-injected or *nr2e3* morphants/*nr2e3* mRNA-injected embryos; mean±s.e.m. and number of samples (*n*) are shown. ****P<0.0001, *P*>0.05 (n.s.), one-way ANOVA with Dunnett's post-hoc analysis.

To improve our understanding of how Flh inhibits parapineal specification, we took a candidate gene approach and identified the orphan nuclear receptor transcription factor Nr2e3 (Nuclear receptor subfamily 2, group E, member 3) as a gene potentially involved in cell-type specification in the pineal complex. In the retina, Nr2e3 opposes Tbx2b function during cell-type specification (Alvarez-Delfin et al., 2009). Similar to *tbx2b* and *flh*, *in situ* hybridization experiments revealed that *nr2e3* is expressed in the epithalamus during pineal and parapineal development (Fig. 2). Thus, the antagonistic relationship of Tbx2b and Nr2e3 may be conserved during pineal complex development. Based on its role in the retina, we tested whether Nr2e3 promotes pineal rod photoreceptor specification. Splice site- and translation initiation-blocking morpholinos were used to eliminate Nr2e3 function at concentrations that significantly decreased Nr2e3 protein levels but caused no overall morphological defects (Fig. 6; Fig. S2A-C; data not shown). Depletion of Nr2e3 caused a reduction in the number of pineal rod photoreceptors (Fig. 6; Table 2; Table S1). However, other non-migratory cell types in the pineal organ were unaffected (Fig. S2D; Table 2). Conversely, we did not observe an increase in the number of rod outer segments (Rhodopsin) or Nr2e3-expressing cells in the pineal organ after overexpression of *nr2e3* mRNA (Fig. 6B). Similar to its role in the retina, our findings demonstrate that Nr2e3 is required for pineal rod photoreceptor specification.

**Table 2.**
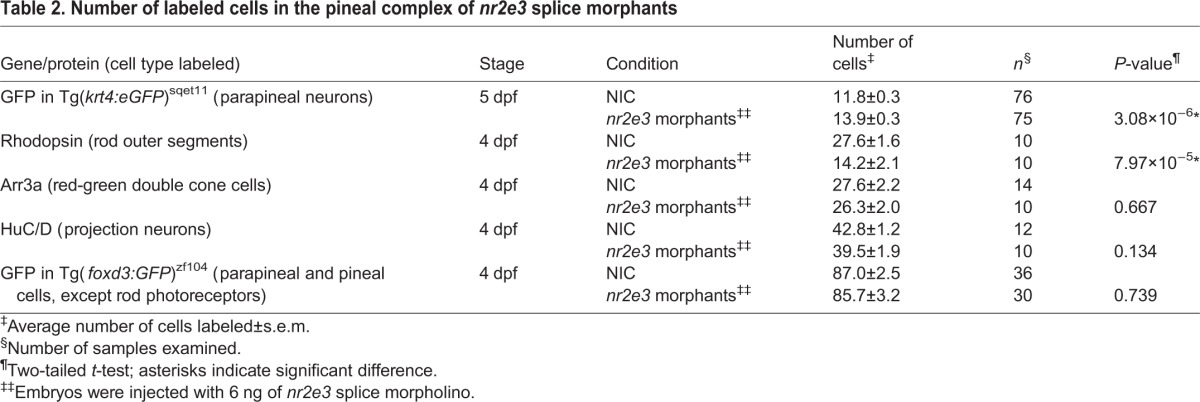
**Number of labeled cells in the pineal complex of *nr2e3* splice morphants**

### Flh inhibits parapineal specification in part by activation of Nr2e3 expression

**Fig. 7.**
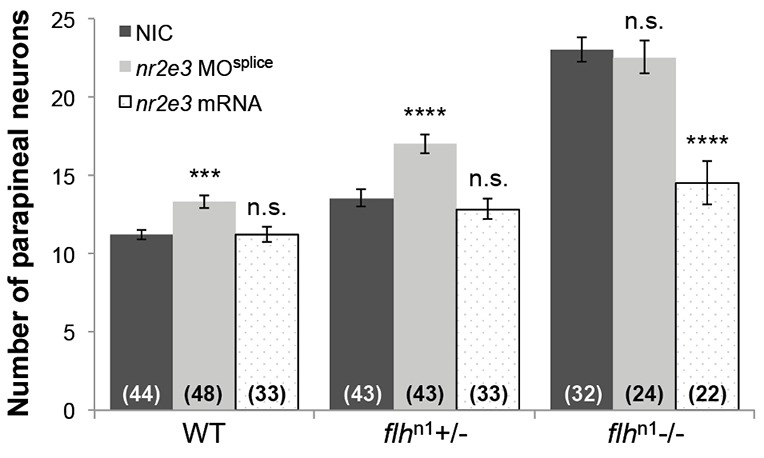
**Flh inhibits parapineal neuron specification through Nr2e3.** Quantification of the number of *krt4*:eGFP-positive cells (parapineal neurons) in WT, *flh*^n1+/−^, and *flh*^n1−/−^ larvae that were either non-injected (NIC), injected with *nr2e3* splice morpholinos (*nr2e3* MO^splice^) or injected with *nr2e3* mRNA; mean±s.e.m. and number of samples (*n*) are shown. ****P<0.0001, ***P<0.001, *P*>0.05 (n.s.), one-way ANOVA with Dunnett's post-hoc analysis.

Importantly, we observed a small but significant increase in the number of parapineal neurons in *nr2e3* morphants compared with NICs at 5 dpf (*P*<0.001) (Fig. 6A; Fig. 7; Table 2; Table S1). Because Flh and Nr2e3 can both inhibit parapineal fate, we sought to determine whether they act in the same genetic pathway. Fig. 1 and Fig. S3 show that loss of Flh caused a reduction of *nr2e3* mRNA and protein levels in the pineal complex, respectively. Because expression of the pro-neural gene *ascl1a* was unaffected by the loss of Flh (Fig. 1), the decrease in expression of *nr2e3* is not due to loss of the total number of pineal complex cells. Similarly, the total number of pineal complex cells in *flh*^n1+/−^ was only slightly reduced compared with WT (Table 1). This allowed us to make a meaningful comparison of Nr2e3 expression between WT and *flh*^n1+/−^ embryos (Fig. S3). To examine further the relationship between Flh and Nr2e3, we tested whether the increased number of parapineal neurons in *flh*^n1^ mutants could be suppressed by *nr2e3* mRNA overexpression. As shown in Fig. 7, we observed partial suppression of the *flh* supernumerary-parapineal phenotype following overexpression of *nr2e3* mRNA. Although mosaic overexpression of tRFP:Nr2e3 resulted in a reduced number of parapineal neurons (Fig. 5B), *nr2e3* mRNA overexpression in WT embryos did not (Fig. 7; *P*=0.9967). Furthermore, when we tested the effects of a reduction in Nr2e3 expression by morpholino injection, we did not observe a further increase in parapineal number in *flh*^n1−/−^ mutants (*P*=0.9096), but we did observe an increase in parapineal number in *flh*^n1+/−^ larvae (*P*<0.0001) (Fig. 7). Together, these data indicate that Flh and Nr2e3 are in the same genetic pathway, with Nr2e3 acting downstream of Flh to inhibit parapineal fate.

### Tbx2b and Nr2e3 act in parallel during parapineal specification

Next, we performed double knockdown experiments with Tbx2b and Nr2e3 and found that they act in parallel during parapineal specification (Fig. S4). In *tbx2b*^c144^/*nr2e3* morphant larvae, the number of parapineal neurons was partially restored (8.5±3.3, *n*=18) relative to the number in *tbx2b*^c144^ mutants alone (2.6±1.3, *n*=26) (Fig. S4B). These data provide additional evidence that Tbx2b enables parapineal specification by relieving repression mediated by Flh and Nr2e3.

### Tbx2b is required for parapineal migration

Consistent with previous findings, the majority of *flh*^n1^ mutants have parapineal organs that migrate correctly (58.3% that are classified as ‘normal’ or ‘bilateral’; Fig. 4A,C) (Gamse et al., 2003). However, 80% of the *flh*^n1^/*tbx2b* morphant larvae demonstrated a defect in parapineal migration in which the neurons remain near the midline (Fig. 4A,C). The reduced migratory ability in our *flh*^n1^/*tbx2b* morphants compared with *flh*^n1^ supports the idea that normal parapineal migration in *flh*^n1^ larvae is due to residual amounts of *tbx2b* (Fig. 1). Similarly, all parapineal neurons remained near the midline in *tbx2b*^c144^/*nr2e3* morphant larvae (Fig. S4A,C). These data indicate that the migratory ability of parapineal neurons depends on functional Tbx2b.

### Reduced habenular asymmetry is observed in larvae with supernumerary parapineal neurons

**Fig. 8.**
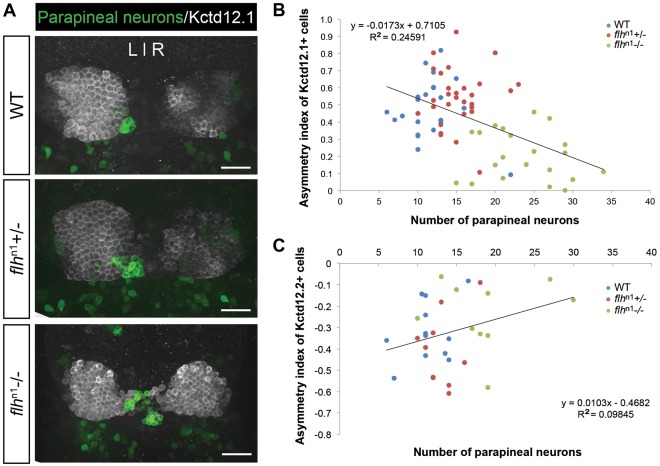
**Decreased habenular asymmetry is observed in Flh-deficient larvae.** (A) Representative images of antibody labeling of *krt4*:eGFP (parapineal neurons) and Kctd12.1 protein (lateral habenular neurons) in the zebrafish epithalamus at 5 dpf. Dorsal views. Scale bars: 30 µm. (B,C) Asymmetry indices of Kctd12.1- (*n*=74; B) and Kctd12.2- (*n*=32; C) positive neurons in Flh-deficient larvae plotted against the numbers of parapineal neurons. The habenulae of larvae with the greatest reduction of Flh (*flh*^n1−/−^), and thus the largest number of parapineal neurons, were more symmetrical compared with their siblings. Pearson's correlation tests were used to determine R^2^ values.

Through an unknown mechanism, the unilateral migration of parapineal neurons profoundly influences habenular asymmetry and function (Concha et al., 2003; Dreosti et al., 2014; Gamse et al., 2003, 2005). However, it is unclear whether the number of parapineal neurons affects habenular development. The supernumerary parapineal phenotype observed in *flh*^n1^ mutants provided an opportunity to investigate how habenular asymmetries are affected by increased parapineal neuron numbers. Using either Kctd12.1 or Kctd12.2 [more highly expressed in the left habenula (lHb) or right habenula (rHb), respectively], as markers, we observed habenular nuclei with reduced left/right asymmetries in *flh*^n1^ mutants (Fig. 8). We calculated asymmetry indices, which represent degrees of asymmetry ranging from 0 (lHb and rHb are completely symmetrical) to 1 (lHb and rHb are completely asymmetrical), and found a weak inverse correlation between the degree of asymmetry and the number of parapineal neurons (R^2^=0.246 and 0.098 for Kctd12.1 and Kctd12.2, respectively; Fig. 8). This suggests that in addition to its proper migration, the size of the parapineal organ is important for asymmetric habenular development. These findings are consistent with a recent study that showed that larvae with reduced habenular asymmetry due to *pitx2c* knockdown have a slightly greater number of parapineal neurons (Garric et al., 2014). Although the increases in parapineal number phenotypes are similar between this study and that of Garric et al. (2014), we found that neither *flh* nor *nr2e3* expression is regulated by Pitx2c (data not shown). Thus, the increased parapineal neuron numbers observed in *flh*^n1^ and *nr2e3* morphants are likely to be independent of Pitx2c.

### The anterior-most domain of the pineal complex anlage is dominated by *tbx2b* expression during parapineal development

**Fig. 9.**
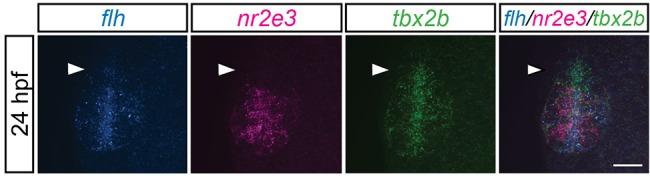
**The anterior-most region of the pineal anlage during parapineal development is mostly *tbx2b*-positive, *nr2e3-* and *flh*-negative.** *tbx2b* expression is expanded in the anterior-most region of pineal anlage (arrowheads) relative to *flh* or *nr2e3* expression patterns. Dorsal views of triple fluorescence DNA hybridization chain reaction *in situ* amplification of *flh*, *nr2e3* and *tbx2b* at 24 hpf. Scale bar: 30 µm.

Spatial regulation of *flh*, *nr2e3* and *tbx2b* might explain how these three genes combine to specify the precise number of parapineal neurons. In order to refine more precisely the expression patterns of these genes during early parapineal development, we performed *in situ* hybridization chain reaction, double fluorescence *in situ* hybridization, and double antibody/fluorescence *in situ* hybridization experiments. We found that *flh* and *nr2e3* were mostly expressed within the same region in the pineal anlage (Fig. 9; Fig. S5A). However, by 24 hpf, the *tbx2b-*positive domain extended anteriorly relative to *flh* (Snelson et al., 2008b) and *nr2e3* (Fig. 9; Fig. S5). Together with previous lineage-labeling experiments (Clanton et al., 2013; Concha et al., 2003), these findings suggest that parapineal precursors are located at the *tbx2b-*positive, *flh/nr2e3-*negative anterior region of the pineal anlage by 24 hpf. It is possible that distinct subpopulations may exist; however, current tools do not allow resolution of Flh-positive from Nr2e3-positive cells in the posterior domain.

## DISCUSSION

In this study, we investigated the role of Tbx2b in the zebrafish epithalamus and found that it is required for (1) parapineal migration and (2) preventing repression of parapineal fate during specification. Our data are consistent with a model in which Flh and Nr2e3 antagonize Tbx2b during parapineal specification. We determined that Flh inhibits parapineal specification, via a mechanism consistent with downstream activation of Nr2e3. Additionally, we found that Flh promotes specification of non-migratory pineal neuron subtypes. Using double knockdown approaches, we showed that parapineal specification and migration could be uncoupled. Lastly, we found reduced habenular asymmetry in larvae with increased numbers of parapineal neurons. Together, our data show that a transcription factor network exists to ensure the proper number and migratory ability of parapineal neurons. Teasing apart the mechanisms of this transcription factor network may provide insights into how cell-fate decisions in other regions of the brain are governed.

### Flh, Nr2e3 and Tbx2b coordinate to govern cell fate in the pineal complex

We propose that proper numbers of non-migratory pineal cell types and migratory parapineal neurons are achieved through a balance of cell-fate specification and inhibition. We believe this is achieved in part through spatial regulation of gene expression within the pineal complex. Expression of Flh and Nr2e3, which inhibit parapineal cell fate, is more posteriorly restricted in the pineal anlage compared with expression of Tbx2b. As a result, only Tbx2b-positive cells in the anterior-most region of the pineal anlage, devoid of inhibition from Flh and Nr2e3, are competent to become parapineal neurons capable of migrating to their final positions, adjacent to the habenular nucleus. This is consistent with previous lineage-labeling experiments demonstrating that by 24 hpf, parapineal precursors are located in the anterior region of the pineal anlage (Clanton et al., 2013; Concha et al., 2003). How the anterior region of pineal anlage becomes Tbx2b positive and Nr2e3/Flh negative is not known. One possibility is that cells destined to become parapineal precursors downregulate Flh and Nr2e3, leaving only Tbx2b to promote specification and migration of parapineal neurons. A similar mechanism has been observed during cell-type specification in the zebrafish retina where Nr2e3 is initially expressed in all photoreceptor precursors, but is later downregulated in cone photoreceptors (Alvarez-Delfin et al., 2009). Alternatively, Tbx2b-positive parapineal precursors, responding to an unknown pro-parapineal signal(s) emanating from the anterior diencephalon, may migrate out from a mixed population of the pineal anlage where they differentiate. Earlier markers of parapineal precursors are needed in order to distinguish between these two possibilities.

Previously, we assumed that Tbx2b could directly specify parapineal fate as very few parapineal neurons are seen in Tbx2b mutants (Snelson et al., 2008b). However, we now propose that this is due to the presence of Flh and Nr2e3. The supernumerary parapineal phenotypes in Flh mutants and *nr2e3* morphants support this hypothesis. Also, mosaic overexpression of Tbx2b cannot induce additional parapineal neurons (Fig. 5). As Tbx2b has been shown to be important for parapineal specification (Snelson et al., 2008b) and Flh positively regulates *tbx2b* expression (Fig. 1), these findings seem counter-intuitive. However, our findings can be understood as negative feedback of Tbx2b to limit Flh inhibition of parapineal formation. Indeed, parapineal neurons can form in the absence of Tbx2b if Flh- or Nr2e3-mediated inhibition is relieved. We observed near-wild-type numbers of parapineal neurons in Tbx2b/Flh and Tbx2b/Nr2e3 double knockdown conditions (Fig. 4B; Fig. S4B). In the absence of Flh or Nr2e3, Tbx2b is able to influence the fate of progenitors in the posterior region and promote the formation of additional parapineal neurons. It is important to note that although Nr2e3 appears to inhibit Tbx2b's function, it does not inhibit Tbx2b itself: we saw no change in *tbx2b* expression in *nr2e3* morphants (Fig. S2E).

### Roles of Flh and Nr2e3 in pineal complex development

Previously, Flh was thought to only control pineal neurogenesis and play no role during parapineal development (Snelson et al., 2008a). Here, we show that Flh, and to a lesser degree, Nr2e3, can negatively regulate parapineal cell fate. We hypothesize that the supernumerary parapineal neurons in Flh mutants and *nr2e3* morphants arise from a more posterior pineal complex anlage. To show conclusively that pineal complex anlage cells that would normally give rise to pineal photoreceptors or projection neurons are becoming parapineal neurons in Flh mutants or *nr2e3* morphants, a detailed cell-fate analysis will be required but the tools necessary to label specific pineal anlage precursors selectively and the necessary markers to distinguish pineal from parapineal neurons in Flh mutants must first be created. Likewise, mosaic overexpression of Flh or Nr2e3 results in fewer parapineal neurons, suggesting that they might directly inhibit parapineal fate and promote other pineal cell types but resolution of this question awaits further tool development.

The importance of Flh during pineal complex development has long been known (Cau and Wilson, 2003; Masai et al., 1997; Snelson et al., 2008a). However, the identities of other factors that act in parallel or downstream of Flh to fine-tune different neuronal subtypes have been elusive. We have found that Nr2e3 functions downstream of Flh in parapineal neuron and pineal rod photoreceptor specification. The expression of Nr2e3 is drastically reduced in Flh mutants. Additionally, whereas all non-migratory pineal cell types are reduced in Flh mutants, loss of Nr2e3 only reduces pineal rod photoreceptors. This suggests that Nr2e3 is specifically required for pineal rod photoreceptor development, echoing its role in the retina (Chen et al., 2005). This finding also suggests that many mechanisms that govern cell-type specification in the retina have analogs in the pineal complex. Identification of other Flh downstream target genes, perhaps via chromatin immunoprecipitation-sequencing analysis, could elucidate how other pineal complex cell types are specified.

### How does Tbx2b drive parapineal formation?

The specification of parapineal neurons appears to be a multi-stage process involving several factors, with Tbx2b governing their competency during early stages of pineal complex development. In the absence of Fgf signaling, parapineal precursors preferentially adopt a red-green cone photoreceptor fate (Clanton et al., 2013). This adoption of cone fate by parapineal precursors is prevented by loss of Tbx2b (Clanton et al., 2013). Here, we showed that Tbx2b is required but not sufficient for parapineal specification. Mosaic overexpression of Tbx2b in pineal complex precursors failed to produce more parapineal neurons (Fig. 5). In the absence of functional Tbx2b, we still observed near-wild-type numbers of parapineal neurons when inhibition of parapineal fate was lessened by loss of Flh or Nr2e3 (Fig. 4B; Fig. S4B). Together, these data support the idea that Tbx2b might regulate the competency of precursor cells to differentiate as parapineal neurons but does not directly assign parapineal fate. This may indicate that another pro-parapineal factor(s) exists or there could be a default number of parapineal neurons, and the function of Tbx2b, Flh and Nr2e3 is simply to fine-tune this specification.

Although the role of Tbx2b during specification of parapineal neurons appears to be indirect, Tbx2b is absolutely essential for migration of parapineal neurons. In the absence of Tbx2b, parapineal neurons in the vast majority of larvae (>80%) fail to migrate away from the midline. Because *tbx2b* expression is reduced in Flh mutants, we were intrigued to discover that parapineal migration is relatively normal in Flh mutants. This might be due to residual amounts of *tbx2b* remaining in Flh mutants; if so, Tbx2b is a very potent factor for parapineal migration. Elucidation of Tbx2b-responsive genes will enable a better understanding of how these parapineal neurons are able to migrate precisely to their target destination. Transcriptome analysis between wild-type and *tbx2b* morphant pineal complexes at 24 hpf identified several differentially expressed genes (Khuansuwan and Gamse, 2014). Further investigation of these differentially expressed genes should provide insight into how Tbx2b regulates parapineal migration.

### Parapineal organ size could influence the degree of habenular asymmetry

In addition to producing the correct numbers of pineal complex cells, the transcription factor network we have identified also exerts an influence on the neighboring habenular nuclei. In wild-type larvae, the left habenula has a much larger Kctd12.1 expression domain than the right habenula, yielding a distinctive left/right asymmetry (Concha et al., 2003; Doll et al., 2011; Gamse et al., 2003). However, Flh mutants exhibit reduced habenular asymmetry as a result of an increase in the Kctd12.1 expression domain in the right habenula (Fig. 8; data not shown). This left-habenular-isomerism phenotype in Flh mutants is similar to that of Pitx2c mutants, which, interestingly, also demonstrate an increased number of parapineal neurons (Garric et al., 2014). It is also important to note that this left isomerism phenotype in Flh mutants is not a result of a notochord defect, as others have reported no reduction in habenular asymmetry in *ntl* morphants, which also lack the notochord (Concha et al., 2003; Gamse et al., 2003). By contrast, right-habenular isomerism (reduced Kctd12.1 expression domain in the left habenula) is observed in Tbx2b mutants, which have greatly reduced numbers of parapineal neurons (Snelson et al., 2008b). The differences in the habenular phenotype between Flh and Tbx2b mutants could result from increased versus decreased numbers of parapineal neurons. Normal habenular asymmetry may require a specific number of parapineal neurons, neither too many nor too few. In most *flh* larvae that exhibit the left-habenular-isomerism phenotype, the enlarged parapineal organs project their axons into either the left or right habenula. This suggests that instead of influencing habenular asymmetry via direct innervation, parapineal neurons might secrete a paracrine signal that promotes habenular neurogenesis.

### The zebrafish pineal complex as a model in which to study neuronal fate decisions

Formation of the brain requires specification of the correct numbers and types of neurons with proper migration and dendritic connections. In the vertebrate retina, a specific birth order specifies the different cell types before ordering into a laminar structure with precise connectivity (for a review, see Cepko, 2014). The same developmental problem exists in the pineal complex: the number of photoreceptors must be accurately specified and must form connections via their associated projection neurons to link the pineal complex with other areas of the brain. At the same time, parapineal neurons emerge and migrate from the same anlage. Thus, the pineal complex provides an accessible, relatively simple model system in which to study key aspects of developmental neurobiology. Identification of transcription networks similar to Tbx2b/Flh/Nr2e3 in the pineal complex will be key to a broader understanding of specification and migration in other regions of the central nervous system.

## MATERIALS AND METHODS

### Zebrafish strains and maintenance

Zebrafish were raised at 28.5°C on a 14 h light/10 h dark cycle. Embryos and larvae were obtained from natural matings and staged according to somite stage (ss), hours post-fertilization (hpf), or days post-fertilization (dpf). The following lines were used: AB* (Walker, 1999), *tbx2b*^c144^ (Snelson et al., 2008b), Tg[*foxd3:gfp*]^zf104^ (Gilmour et al., 2002), *flh*^n1^ (Talbot et al., 1995), Tg[*cfos:gal4vp16*]^s1145t^ (Scott and Baier, 2009) and Tg[*krt4:egfp*]^sqet11^ (Parinov et al., 2004)*.* All experiments were approved by the Vanderbilt University's Institutional Animal Care and Use Committee (IACUC) and Office of Animal Welfare, and performed according to national regulatory standards.

### *In situ* hybridization

Whole-mount chromogenic RNA *in situ* hybridizations were performed as previously described (Gamse et al., 2003). Whole-mount fluorescence RNA *in situ* hybridizations were performed as previously described with the addition of 5% dextran sulfate (Sigma) to enhance the staining (Lauter et al., 2011). Information on RNA probes is listed in Table S2. For detailed protocols, see supplementary Materials and Methods.

### DNA hybridization chain reaction *in situ* amplification

Whole-mount DNA hybridization chain reaction (HCR) *in situ* amplification was performed as previously described (Choi et al., 2014). Two-initiator DNA probes for *flh*, *nr2e3* and *tbx2b* and DNA HCR amplifiers (Molecular Instruments) were utilized. Information on probes and amplifiers is listed in Table S3.

### Immunofluorescence

Samples for whole-mount immunofluorescence labeling were fixed overnight at 4°C in 4% paraformaldehyde (PFA) and processed as described (Snelson et al., 2008b). Information on the primary and secondary antibodies is in Table S4. To visualize cell nuclei, samples were incubated with ToPro3 (T3605, Invitrogen, 1:10,000). Fluorescence *in situ* hybridization/immunofluorescence double labeling was performed as described (Doll et al., 2011).

### Imaging

For chromogenic *in situ* labeling, brightfield images were obtained from glycerol-cleared embryos using Qcapture software (QImaging) with a Retiga EXi Fast 1394 cooled monochrome-12 bit CCD camera (QImaging) attached to a RGB color filter (QImaging) mounted on a Leica DM6000B microscope with a 20× objective. For all fluorescence labeling, glycerol-cleared embryos or larvae were imaged from dorsal views on a PerkinElmer RS3 spinning disk confocal microscope with a 40× oil-immersion objective. Images were analyzed with Volocity software (Improvision).

### Morpholino injections

Embryos were injected with 1 nl volumes of morpholino antisense oligonucleotides diluted in nuclease-free water at the one-cell stage. The following morpholinos and concentration were used in this study: 6 ng nl^−1^ *tbx2b* splice-blocking morpholino (*tbx2b* MO^splice^), 5′-AAAATATGGGTACATACCTTGTCGT-3′ (Snelson et al., 2008b); 6 ng nl^−1^ *nr2e3* splice-blocking morpholino (*nr2e3* MO^splice^), 5′-ATACGCAAGTTGTTTTCTCACCTGT-3′ (complementary to the exon 2/intron 2 junction); 8 ng nl^−1^ *nr2e3* translation initiation-blocking morpholino (*nr2e3* MO^ATG^), 5′-GATCCTCCATTGAAGGTGGTGTAAA-3′ (complementary to a region encompassing both predicted start sites); 2 µM *ntl* translation initiation-blocking morpholino (*ntl* MO) (Nasevicius and Ekker, 2000); 1.5 mM *pitx2c* translation initiation-blocking morpholino (Garric et al., 2014).

### Mosaic overexpression

One- to four-cell-stage Tg[*foxd3:gfp*]^zf104^; Tg[*cfos:gal4vp16*]^s1145t^ double transgenic embryos were injected with 1 nl solution of UAS-containing plasmid DNA (see supplementary Materials and Methods) at a concentration of 25 ng µl^−1^, Tol2 transposase RNA at 25 ng µl^−1^, and Phenol Red at 0.05%. Tg[*cfos:gal4vp16*]^s1145t^ drives expression of Gal4 transcription factor in the pineal complex by 12 ss (Corey Deanne Snelson, PhD thesis, Vanderbilt University, 2009). Injected embryos were raised at 28.5°C until 48 hpf when they were fixed in 4% PFA overnight at 4°C for antibody labeling of GFP and tRFP-T. Parapineal neurons were counted from embryos that expressed five to fifteen tRFP-, tRFP:Tbx2b-, Flh:tRFP- or tRFP:Nr2e3-positive cells in the pineal complex.

### *nr2e3* mRNA overexpression

*nr2e3* mRNA was transcribed *in vitro* using the mMessage mMachine transcription kit (Ambion) from the pRK5-*nr2e3* template (linearized with *Hpa*I, transcribed with SP6 polymerase) (Chen et al., 2005). mRNA was injected or simultaneously co-injected with *nr2e3* splice-blocking morpholino into one-cell-stage embryos as indicated.

### Semi-quantitative RT-PCR

RT-PCR for *nr2e3* was performed on total RNA isolated from embryos or larvae with Trizol according to the manufacturer's instructions (Invitrogen). Reverse transcription was performed with random hexamer primers, followed by PCR amplification using the following primers: 5′-TCCTGAACACGGGACTTCTT-3′ and 5′-TTCAGCTTGAAGGCATTTCT-3′.

### EdU labeling

EdU labeling of dechorionated Tg[*foxd3:gfp*]zf104 embryos was performed using the Click-it Alexa Fluor 647 Imaging Kit (Life Technologies, C10340). For detailed protocols, see supplementary Materials and Methods.

### Parapineal migration

Parapineal-migration phenotypes were determined based on the position of parapineal neurons relative to acetylated tubulin, as well as the direction of parapineal neuron projections. When two parapineal organs were observed, parapineal migration phenotypes were assessed for each parapineal organ and classified as mixed (when only one of two parapineal organs migrated correctly) or bilateral (when one of the parapineal organs migrate to the left and the other to the right of the pineal organ).

### Habenular asymmetry index

To determine the asymmetry index (AI) for an individual larva fluorescently labeled with either Kctd12.1 or Kctd12.2 antibodies, the volume (determined by fluorescence intensity) of parapineal-adjacent habenula (L) minus the volume of the opposite habenula (R) was divided by the total volume of both habenulae, i.e. AI=(L−R)/(L+R) (Roussigne et al., 2009). AI was then plotted against the corresponding number of parapineal neurons of the same larva.

### Statistics

Two-tailed Student's *t*-tests were used to compare between two groups. One-way ANOVA tests were used for comparisons among three or more groups followed by Tukey's multiple comparisons tests or Dunnett's multiple comparisons tests, as indicated. Pearson's correlation tests were used to determine R^2^ values.
